# Review of the Use of Animal Models of Human Polycystic Kidney Disease for the Evaluation of Experimental Therapeutic Modalities

**DOI:** 10.3390/jcm12020668

**Published:** 2023-01-14

**Authors:** Shizuko Nagao, Tamio Yamaguchi

**Affiliations:** 1 Advanced Research Center for Animal Models of Human Diseases, Fujita Health University, Toyoake 470-1192, Japan; 2 Department of Medical Technology, Faculty of Health Science, Suzuka University of Medical Science, Suzuka 510-0293, Japan

**Keywords:** cystic kidney, animal model, spontaneous, mutation, gene targeting

## Abstract

Autosomal dominant polycystic kidney disease, autosomal recessive polycystic kidney disease, and nephronophthisis are hereditary disorders with the occurrence of numerous cysts in both kidneys, often causing chronic and end-stage renal failure. Animal models have played an important role in recent advances in research not only on disease onset and progressive mechanisms but also on the development of therapeutic interventions. For a long time, spontaneous animal models have been used as the primary focus for human diseases; however, after the identification of the nucleotide sequence of the responsible genes, *PKD1*, *PKD2*, *PKHD1*, and *NPHPs*, various types of genetically modified models were developed by genetic and reproductive engineering techniques and played the leading role in the research field. In this review, we present murine models of hereditary renal cystic diseases, discussing their potential benefits in the development of therapeutic strategies.

## 1. Introduction

Recently, research on hereditary renal cystic diseases such as polycystic kidney disease (PKD) and nephronophthisis (NPHP) has exponentially progressed, facilitating the ongoing identification of genetic variants that lead to the development of adult PKD with the use of laboratory animals [1]. This review focuses on the utility of laboratory rodent models in the development of treatment strategies for renal cystic diseases.

PKD, a typical hereditary disease of the kidney, is classified based on the responsible gene and mode of inheritance [2]. In adults, autosomal dominant PKD (ADPKD) is the most common inherited renal disorder [3]. In addition to the involvement of both the kidneys, cysts frequently develop in the liver as well as the pancreas, and systemic hypertension and cerebral aneurysms are common comorbid conditions [4]. Clinical manifestations commonly begin between the ages of 30 and 50 years, and by the age of 70 years, nearly 50% of patients develop end-stage renal disease [5]. Approximately 85% of patients who develop ADPKD are found to have mutations in *PKD1*, whereas the remaining 15% have mutations in *PKD2* [6]. The pathological conditions caused by *PKD1* mutations have been reported to progress faster than those caused by *PKD2* [7]. With regard to *PKD1* mutations, patients with truncated mutations have been reported to progress faster than those with nontruncated mutations [8]. This contrasts with autosomal recessive PKD (ARPKD), which is caused by *PKHD1* mutations and characterized by enlarged renal collecting ducts and congenital hepatic fibrosis [9]. The onset of clinical symptoms of ARPKD typically occurs in the fetal or neonatal period or, at times, as late as early childhood [10]. Prenatal diagnosis with immediate transfer of the neonate to a neonatal intensive care unit improves the survival rate to at least 1 month and is associated with survival up to the age of 5 years in 85% of babies who survive past the first 30 days [11].

*PKD1* and *PKD2* encode Polycystin-1 (PC-1) and Polycystin-2 (PC-2/TRPP2), respectively. PC-1, which functions as a mechanosensor of intraluminal fluid flow, interacts with PC-2/TRPP2, which has calcium (Ca^2+^) channel activity in its C-terminus [12]. The *PKHD1* gene product fibrocystin/polyductin (FPC) has a long extracellular domain and interacts with the N-terminus of PC-2 [13].

Approximately 5–25% of cases of premature renal failure are directly attributable to NPHP, a disease caused by *Nphp* mutations and characterized by the presence of numerous cysts in the renal medulla that is typically detected during childhood [14]. ADPKD, ARPKD, and NPHP are referred to as “ciliopathies” because of the presence of their gene products in primary cilia. The exacerbation of their associated pathological conditions results in increased cellular proliferation, inflammation, fibrosis, and accumulation of cystic fluid [15].

To elucidate the pathogenesis of PKD and test the efficacy of the candidate drugs, spontaneous and genetically modified laboratory animal models are the primary source of development information. The revelation of the nucleotide sequences of *PKD1*, *PKD2*, and *PKHD1* has led to the development of various genetically modified animals, including transgenic animals with a foreign gene inserted, knockout (KO) animals with a specific gene disrupted, conditional KO (CKO) animals with cells forming a specific organ intentionally damaged or genetically disrupted at specific locations, knock-in animal models with hypomorphic genetic mutations, and genome-editing animal models that have been modified using new techniques such as the CRISPR/Cas9 method [16,17,18,19]. This study introduces the use of animal models in research on renal cystic disease and characterizes different animal model strains.

## 2. Spontaneous Model Animals

Spontaneous renal cysts in model animals were identified in the early 1980s before the discovery of the responsible genes. *Cys1*, *Nphp3*, *Anks6* (also called *Pkdr1/Nphp16*), and *Pkhd1* have since been identified as responsible for the development of renal cysts in murine strains in the cpk mouse, pcy mouse, Han:SPRD-*Cy*/*+* rat (commonly known as the Cy rat), and PCK rat, respectively. Their gene products were reported to be present in cilia proteins such as PC-1, PC-2, and FPC, which are the products of the expressions of ADPKD genes *Pkd1* and *Pkd2* and the ARPKD gene *Pkhd1*, respectively [20]. The characteristics of the aforementioned spontaneous animal models are listed below.

### 2.1. cpk Mice

Renal disorders in cpk mice are caused by a mutation in *Cys1*. The gene product, cystin, regulates *myc* expression through its interaction with the tumor suppressor necdin. *Cys1* was confirmed to be orthologous mouse gene for humans with ARPKD [21]. A study has also been reported that M2-like macrophages from cpk mice contribute to the progression of cystic cell proliferation, cyst growth, and development of fibrosis. Treatments that block the emergence of these types of immune cells are thought to be emerging medications that prevent disease progression [22].

### 2.2. pcy Mice

**Figure 1:**
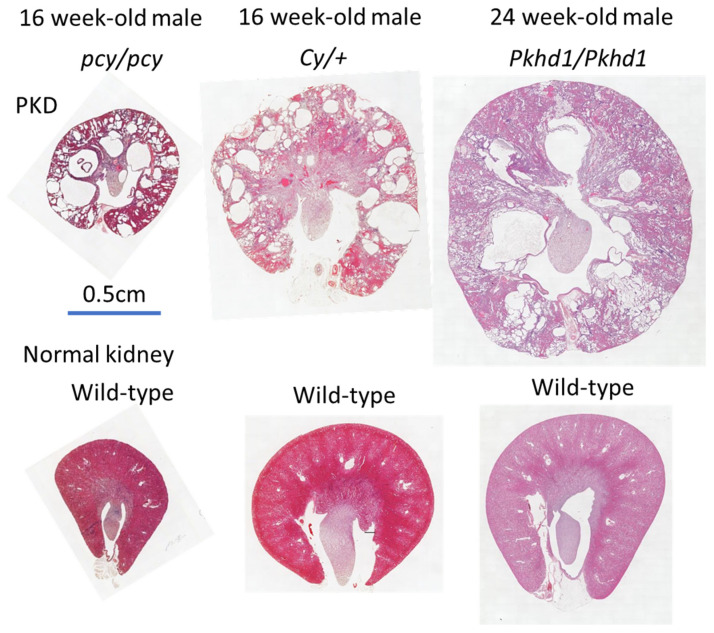
Macrophotographs in the hematoxylin and eosin-stained kidneys of pcy mice and Cy rats and the PAS-stained kidneys of PCK rats.

The dilation of the distal tubules along the corticomedullary junction leads to the formation of renal cysts throughout the nephron in pcy mice (Figure 1) [23]. Disease progression in pcy mice, which occurs more rapidly in females than in males, is relatively gradual, leading to a shortened lifespan of 40–50 weeks secondary to renal failure, compared with up to 3 years in native mice [24]. The renal pathology is caused by the missense mutation (T1841G) of *Nphp3* on chromosome 9 [25]. Consequently, the production of cyclooxygenase (COX) increases, whereas that of lipoxygenase (LOX) decreases in the kidneys of pcy mice, suggesting that altered eicosanoids are involved in NPHP progression. Therefore, the manipulation of these levels and balances with pharmaceutical drugs may have potential therapeutic benefits [26]. A recent report showed that a phosphate-restricted diet in pcy mice increased the serum levels of calcium and expression levels of *Klotho*, inhibited renal cyst formation, and activated the signaling cascades involved in the development of renal fibrosis [27].

### 2.3. Cy Rats

In Cy rats, the missense mutation in *Anks6* on chromosome 5 causes an R823W substitution of SamCystin, the native gene product, which results in a renal cystic disorder [28,29]. The phenotype exhibits incomplete dominance in homozygotes (*Cy/Cy*), which are known to rapidly develop renal cysts and cause death by ~3 weeks of age [30]. Heterozygotes (*Cy/+*) exhibit a slower disease progression, with an average lifespan of ~1 year in males and 18 months in females (Figure 1) [25]. Recent studies have shown that a 3D capsule device overlaying the entire kidney of a *Cy/+* rat reduced the kidney weight, size of the renal cysts, and proliferation rate of both cystic epithelial cells and macrophage infiltration [31].

### 2.4. PCK Rats

PCK rats develop renal and hepatic disorders because of the deletion of the *Pkhd1* exon 36, which is orthologous to the human gene responsible for ARPKD [32,33]. The PCK rats develop Caroli’s disease, which is characterized by the dilation of the intrahepatic bile ducts and hepatic fibrosis and cysts derived from the collecting ducts, which spread throughout the renal nephrons (Figure 1) [34]. The lifespan of females is over 6 months longer than the 18-month lifespan of males [25]. Tolvaptan, an arginine vasopressin (AVP) V2-receptor antagonist developed in Japan, has shown beneficial effects on PCK rats [35]. Subsequently, it has been approved in many countries and has become the world’s first drug used to treat ADPKD [36]. Previous studies have also reported that increasing water intake significantly ameliorated disease progression in PCK rats by reducing AVP secretion into the blood from the posterior pituitary gland [37].

## 3. Genetically Modified Models

The goal of research in genetically mediated diseases is to determine whether its manifestation is caused by the gain or loss of function caused by the expression of the mutation. Transgenic models are useful when gain-of-function is the etiology, whereas KO or gene-targeting models are useful when renal disorders are caused by loss of function, with gene mutations occurring in all cells from the embryonic and fetal stages [38,39].

### 3.1. KO and CKO Mice

Owing to embryonic lethality in KO homozygous mice, CKO animals were developed with induced mutations limited to specific tissues/cells or introduced at controlled times during organ development. In some CKO animals [40,41,42], it is possible to control the rate of disease progression and cause gene disruption in specific organs through flox mice, which have a gene locus flanking the target region using the Cre-loxP system, and mating with Cre-expressing mice [43].

In ADPKD CKO model mice presenting with mitochondrial dysfunction, the administration of MitoQuinone, a mitochondrion-specific antioxidant, caused the inactivation of ERK/MAPK signaling, reduction of intracellular superoxide levels, and inhibition of cellular proliferation in cystic epithelia [44]. Further, in CKO model mice, the administration of Mdivi-1, a mitogenic protein DRP-1 inhibitor, suppressed disease progression [45].

By contrast, the Yes-associated protein (YAP) was identified as a key transcription factor in the Hippo signaling pathway [46]. CKO mice showed an increase in actomyosin contraction, YAP nuclear translocation, and YAP transcriptional activity [47]. Fasudil reversed YAP activation and suppressed renal disease progression [48]. In other CKO mice, reducing YAP expression with an antisense oligonucleotide did not ameliorate renal cystic disease; however, it did overexpress the downstream targets of the WNT and TGF-β pathways, Myc, Acta2, and Vim [49]. Further studies on the role of YAP in PKD progression deserve serious consideration.

Cyclic adenosine monophosphate (cAMP) is known to mediate cell proliferation and fluid secretion of renal cystic epithelia in PKD [50]. In the CKO mouse, the administration of a pharmacological inhibitor of the cAMP response element binding protein (CREB) and a dominant-negative inhibitor of CREB suppressed renal cystic area expansion [51]. The reduction of the intracellular cAMP concentration by SR59230A, a selective β3-adrenergic receptor antagonist, suppressed renal cystic disease progression in other CKO mice [52].

Macrophage recruitment and interstitial inflammation are involved in the exacerbation of PKD pathology by promoting cystic growth. Tumor necrosis factor-like weak inducer of apoptosis (TWEAK) and its receptor, the fibroblast growth factor, inducible 14 (Fn14), were expressed in the macrophages of affected mice [53]. Their overexpression was detected in two kinds of CKO mice, in which the administration of TWEAK led to the exacerbation of PKD, and treatment with anti-TWEAK antibodies ameliorated PKD, resulting in improved survival [54].

Interferon regulatory factor-5 (IRF5) is a transcription factor associated with renal cyst-promoting cytokines in macrophages. An injection of the IRF5 antisense oligonucleotide into CKO mice reduced the number of macrophages, IRF5 expression in the identified macrophages, and renal cystic disease severity [55].

### 3.2. Double-Mutant Mice

Several studies have used double-mutant models in which a target gene is mutated other than the CKO gene responsible for PKD. In this regard, the *Pkd1^RC/RC^ Pkd2^+/−^* mouse presenting with congenital renal cysts shows moderate renal disease progression, where cysts continue to grow proportionally with the ferritin level [56]. Treatment with ciclopirox olamine salt, an antifungal agent, reduced renal cyst progression and improved renal function by decreasing the ferritin level [57].

In another *Pkhd1-Cre; Pkd2^flox/flox^; miR-214^−/−^* model mouse, increased accumulation of pericystic macrophages was detected with an upregulation of the inflammatory TLR4/IFN-γ/STAT1 signaling pathway [58]. miR-214, a microRNA, restrained cyst-associated inflammation (by inhibiting TLR4) and attenuated cyst growth [59]. In a study aimed at elucidating the effect of microRNA, the administration of RGLS4326, an anti-miR-17 oligonucleotide, suppressed the progression of PKD in CKO mice [60].

The procedure for renal fibrosis suppression in double-mutant PKD models with YAP gene deletion or renal collecting duct-specific gene CCN2 deletion has included the administration of verteporfin, the YAP inhibitor that is known to have an inhibitory effect on the fibrotic process [61].

### 3.3. Knock-in Mice

A knock-in model with hypomorphic alterations in the mitochondrial morphology was identified in *Pkd1^RC/RC^* and *Pkd1^null/RC^* mice, corresponding to a disease variant in the ADPKD phenotype [62,63]. Recently, these strains have been used in the experimental administration of drugs affecting the signaling pathways altered by *Pkd1* mutations. The following drugs showed ameliorative effects on renal disease progression (Table 1): AMPK activators, metformin [64], lixivaptan (a novel AVPR2 antagonist), with R-568 (a calcium receptor agonist for a combined inhibitory effect) [65], BLU2864, a PKA inhibitor [66], a CFTR-trafficking and processing modulator, VX-809, [67], nintedanib, a tyrosine kinase inhibitor [68], and a combination treatment with tolvaptan (an AVPV2 receptor antagonist), and pasireotide (a somatostatin analog) [69].

### 3.4. Genome Editing

Recently, model animals have been created using genome-editing techniques such as CRISPR/Cas9 [87]. For instance, in the Pkhd1^C642*^ mouse with a predicted truncating mutation in the middle of exon 20, a cluster of five truncating human mutations between *Pkhd1*^G617fs^ and *Pkhd1*^G644*^ was generated. In the heterozygous Pkhd1^C642*^ mouse, hepatic cysts developed, whereas in the homozygous mouse, congenital hepatic fibrosis, inflammation of the portal field, and fibrosis manifestations developed, suggesting that the heterozygous *PKHD1* mutation might cause cystic liver disorders [85]. Another gene-editing model in *Nphp1^del2−20/del2−20^* mice mimicked human NPHP, with the development of renal cysts, thickening of the tubular basement membrane, retinal degeneration, and abnormal spermatogenesis. An adenoviral-associated-virus-9 vector was noted to partially rescue both renal and retinal phenotypes [86]. Further ingenuity in genome-editing techniques is certainly on the horizon because KO model animals inconsistently reflect the changes seen in human renal cystic diseases.

## 4. Discussion

This review described animal model research for the treatment of human renal cystic diseases, focusing on recently published papers.

For the drug treatment study, we recommend using several kinds of models. Specifically, phenotypically established spontaneous models, such as pcy mice, Cy rats, and PCK rats, which reliably exhibit pathology with relatively long lifespans of more than half a year, are suitable for medicine efficacy experiments. In the KO mouse model, the orthologous gene is always mutated; however, the pathology differs depending on the mutation site. The models’ lifespans are usually either too short or too long. It is suitable for the analysis of cystogenesis rather than medicine efficacy experiments, including preclinical trials. In the CKO model and the hypomorphic allele model, the orthologous gene is always mutated, and the pathology is constant, although it is necessary for a relatively long period. Therefore, it is suitable for both medicine efficacy experiments and analyses of cystogenesis (Pkd1^flox/flox; Ksp-Cre^, Pkd1^RC/RC^).

To determine the efficacy of treatments, kidney volume, kidney-weight-to-body-weight ratio, urine/plasma biomarkers (creatinine, urea nitrogen, L-FABP, etc.), renal cAMP concentrations, and/or histopathological indices (fibrosis index, cyst area, cell proliferation index, and target protein distribution) are important outcome indicators.

In both spontaneous and genetically modified models, disease progression is often variable. Thus, the number of control vehicle animals should not be too small when conducting a preclinical efficacy trial, and each group should include a sufficient number of animals to determine efficacy accurately. The clinical presentation of human and rodent PKD is most pronounced in adulthood. However, it is generally desirable to start preclinical trials during the early stage when there are few clinical features and continue until clinical symptoms appear in the control vehicle group. Both male and female animals should be used because of clinical evidence of gender hormone influence. Because PKD is a kidney disease, the concentration and quality of proteins in chow during the treatment should be considered. Moreover, since animal welfare and humane care are important, animal experiments should follow the ARRIVE 2.0 guidelines (https://arriveguidelines.org/arrive-guidelines, accessed on 1 November 2022).

To increase the credibility of the results, the use of multiple models, including spontaneous and genetically modified models, or mouse and rat models, is effective because the same drug may have both beneficial and adverse effects on different animal models. This indicates the difficulty in determining which model is relevant or not.

Based on our experience with numerous model animals for drug efficacy studies, some of the existing models exhibit disease progression too early and others too late, and some are appropriate as pathological models; however, the responsible gene is not orthologous to human patients. Further efforts are required to develop pathological models of human PKD.

## 5. Summary and Conclusions

Several representative models of renal cystic disease were reviewed in this article; however, it is difficult to exhaustively cover them all. We listed the models with their applications based on recently reported novel findings (Table 1). As mentioned, these models may become useful for the development of new therapeutic options based on the discovery of novel signaling pathways and involve the verification of factors in the exacerbation of PKD. Medications may have variable rates of response and efficacy when administered to different animal models, and the use of multiple animal strains has been proven critical in the development of effective therapeutic options. In this review, we studied animals that spontaneously developed renal cysts and genetically modified animals with their respective benefits. Currently, the number of studies on animal models of renal cystic diseases is increasing exponentially compared with previous decades. We believe that this review will benefit those seeking to understand the utility and assist in the classification of animal models of human renal cystic diseases to make treatment available to all patients.

## Figures and Tables

**Table 1:** Animal models and their applications are based on recently reported novel findings.

Gene	Strain/Gene Name	Treatment/Analysis/ Administration	Results/Effects	References
**Cy Rat**				
*Nphp16*	Han:SPRD-*Cy/+*	3D capsule device	Suppression of the PKD progression.	[31]
*Nphp16*	Han:SPRD-*Cy/+*	MitoQuinone, a mitochondria-specific antioxidant	Inactivation of ERK/MAPK. Reduction of intracellular superoxide. Inhibited proliferation of the epithelial cysts.	[44]
**pcy Mouse**				
*Nphp3*	*Pcy*	RGLS4326, an anti-miR-17 oligonucleotide	Suppression of the PKD progression.	[60]
**PCK Rat**				
*Pkhd1*	PCK	Lixivaptan, a novel V2R antagonist and R-568, a calcium receptor agonist	Decrease in the cAMP level. Suppression of the PKA activity. Decrease in the phosphorylated AMPK and ERK. Suppression of the PKD progression. Suppression of fibrosis.	[67]
**KO and CKO Mice**				
*Pkd1*	*Pkd1^flox/flox^; Ksp-Cre*	MitoQuinone, a mitochondria-specific antioxidant	Inactivation of ERK/MAPK. Reduction of intracellular superoxide. Inhibited proliferation of epithelial cysts.	[44]
*Pkd1*	*Pkd1^flox/−^; Ksp-Cre*	-	Disturbances in mitochondrial structure and function. Decreased expression of the fusion-promoting proteins OPA1 and MFN1. Increased expression of the mitogenic protein DRP1.	[45]
		Mdivi-1, a DRP-1 inhibitor	Suppression of the PKD progression. Improvement of the renal function.	
*Pkd1*	*Pkd1^flox/−^; Ksp-Cre*	-	Increased actomyosin contraction. YAP nuclear translocation. Enhanced YAP transcriptional activity.	[48]
		Fasudil, a protein kinase inhibitor	Inhibition of Rho kinase (ROCK)-dependent actomyosin contraction. Inhibition of YAP activity.	
Pkd1	iKspPkd1 del	Antisense oligonucleotides	Downregulation of YAP, a key transcription factor in the Hippo signaling pathway, but upregulation of downstream targets Myc, Acta2, and Vim, in the WNT and TGF-β pathways.	[49]
*Pkd1*	*Pkd1^fl/fl^; Cre/Esr1^+^*	-	Increase of phosphorylated CREB (p-CREB) and of active histone modifications (H3K4me3 and H3K27ac).	[51]
		666-15, a pharmacological inhibitor of CREB (cAMP response element binding protein)	Inhibition of the expansion of the cystic area.	
	*Pkd1^fl/fl^; Cdh16-Cre*	Genetic inhibition with a dominant-negative inhibitor of CREB (A-CREB)	Inhibition of the cystic area expansion.	
**Gene**	**Strain/Gene Name**	**Treatment/Analysis/** **Administration**	**Results/Effects**	**References**
**KO and CKO Mice continued**				
*Pkd1*	*Pkd1^fl/fl^; Pax8^rtTA^; TetO-Cre*	SR59230A, a selective β3-adrenergic receptor antagonist	Reduction of cAMP concentration. Inhibition of the PKD progression. Partial improvement of the renal function.	[52]
*Pkd1*	*Pkd1^cond/cond^; Tam-Cre2*, *Pkd1^cond/cond^; Tam-Cre1*	-	Overexpression of TWEAK (Tumor necrosis factor-like weak inducer of apoptosis) and Fn14 (fibroblast growth factor-inducible 14).	[54]
		TWEAK (Tumor necrosis factor-like weak inducer of apoptosis) anti-TWEAK	Exacerbation of PKD progression. Suppression of PKD progression. Improvement of the survival rate. Decrease in cell proliferation, NF-κB pathway activation, fibrosis, apoptosis, and macrophage infiltration.	
*Pkd1*	*Pkd1^f/f^; Cre-ER^TM^*	IRF5 (Interferon regulatory factor 5) antisense oligonucleotides	Suppression of PKD progression. Reduced number of macrophages. Reduced homeostasis. Decreased expression of the IRF5 in the macrophages.	[55]
*Pkd1* *Pkd2*	*Pkd1^RC/RC^ Pkd^2+/−^*, *Pkd1^RC/RC^ Pkd2^+/+^*	-	Cystic formation. Elevated ferritin levels.	[57]
		CPX (Ciclopirox; 6-Cyclohexyl-1-hydroxy-4-methyl-2(1H)-pyridone) or its olamine salt (CPX-O)	Suppression of PKD progression. Decreased ferritin levels.	
*Pkd1* *Pkd2*	*Ksp-Cre; Pkd1^fl/fl^*, *Pkhd1-Cre; Pkd2^fl/fl^*, *Pkd1^RC/RC^*, *Pkhd1-Cre; Pkd2^fl/fl^; miR-214^−/−^*	Inhibition of miR-214 (double mutant)	Exacerbation of PKD progression. Increased Tlr4 expression (inflammatory TLR4/IFN-γ/STAT1 signaling pathway). Increased accumulation of the pericystic macrophages.	[59]
*Pkd2*	*Pkhd1-Cre; Pkd2^F/F^*	RGLS4326, an anti-miR-17 oligonucleotide	Suppression of PKD progression.	[60]
*Pkd1*	*Pkd1^f/f^; Pkhd1^cre^*, *Pkd1^f/f^; Yap^f/f^; Pkhd1^cre^*, *Pkd1^f/f^; CCN2^f/f^; Pkhd1^cre^*	Verteporfin, a YAP inhibitor Deletion of YAP gene (double mutant) Deletion of CCN2, a renal collecting duct-specific gene (double mutant)	Inhibition of renal fibrosis.	[61]
*Pkd1*	*Pkd1^f/f^; Pkhd1-Cre*	Nintedanib, which selectively inhibits PDGFR, FGFR, and VEGFR	Suppression of PKD progression. Reduced proliferation of epithelial cysts. Decreased expression of growth factors including YAP.	[68]
*Pkd1*	*Pkd1^fox/^* *^−^* *; Ksp-Cre*	Curcumin and ginkgolide B	Suppression of EGFR/ERK1/2, JNK, PI3K/mTOR, and p38 signaling pathways.	[70]
*Pkd1*	*Pkd1^loxp/loxp^; Ksp-Cre*	Cardamomine nominated from natural product library screening	Inhibition of PKD progression. Inhibition of renal cyst development and interstitial fibrosis.	[71]
*Pkd1*	*Pkd1^fl/fl^; Pkhd1-Cre*	Vorasertib, an inhibitor of Plk1 (Polo-like kinase 1)	Suppression of PKD progression by the antioxidant action pathway of peroxiredoxin 5 (Prdx5)-Polo-like kinase 1 (Plk1).	[72]
*Pkd1*	*Pkd1^fl/fl^; Pax8^rtTA^; TetO-Cre*	Hydroxyfasudil, a ROCK (Rho-associated coiled-coil containing protein kinase) inhibitor	Suppression of PKD progression. Reduction of centrosome RhoGAP (ARHGAP). Suppression of ROCK signaling pathway.	[73]
*Pkd1*	*Pkd1^fl/fl^; Pax8^rtTA^; TetO-Cre*	-	Increased activity of calcium-dependent CAPN (Calpain) protease.	[74]
		CAPN (Calpain) inhibitor	Restoration of lysosomal function. CTSB processing/activity, autophagosome and lysosomal fusion.	
**Gene**	**Strain/Gene Name**	**Treatment/Analysis/** **Administration**	**Results/Effects**	**References**
**KO and CKO Mice continued**				
*Pkd1*	*Pkd1^fox/fox^; Nestincre*, *Pkd1^fox/−^; Nestincre*	Secondhand smoke exposure	Acceleration of PKD progression. Increased tubular cell proliferation and apoptosis. Promotion of renal fibrosis. Reduction of glutathione level. Decreased contractile function and structural parameters in the heart. Noticeable reduction of body weight.	[75]
*Pkd1*	*KspCreERT2; Pkd1^lox/lox^*	-	Increased expression of the transmembrane Protein 16A (TMEM16A) and the cystic fibrosis transmembrane conductance regulator (CFTR). Increase of the cystic area.	[76]
		TMEM16A(transmembrane Protein 16A) antagonists niclosamide and benzbromarone TMEM16A-specific inhibitor Ani9	Inhibition of TMEM16A. Reduced expansion of the cystic area. Suppression of the abnormal proliferation of the epithelial cysts.	
*Pkd2*	*Pkd2^−/−^*	DA1(dopamine receptor 1) antagonist, SCH23390	Suppression of disease progression in PKD. Restored sensitivity of flow-activated Na^+^ and HCO3^−^ transport.	[77]
*Pkd2*	*Pkhd1-Cre; Pkd2^F/F^; miR-21^−/−^*	Inhibition of miR-21 (double mutant)	Suppressed expansion of cyst area by regulating apoptosis and proliferation of epithelial cells, and interstitial inflammation.	[78]
*Pkd1*	*Pkd1^RC/RC^*	-	Increased expression of IGF-1 pathway genes.	[79]
	*Pkd1^RC/RC^; Pappa^+/–^*, *Pkd1^RC/RC^; Pappa^–/–^*	Deletion of PAPP-A (Pregnancy Associated Plasma Protein A) gene (double mutant)	Inhibition of disease progression in PKD.	
*Pkd1*	*Pkd1^flox/flox^*; *Ksp-Cre* *Pkd1^flox/flox^*; *Aqp2-Cre*	-	Increased activity of the focal adhesion kinase (FAK).	[80]
		FAK (focal adhesion kinase) inhibitors (double mutant)	Suppression of PKD progression. Inhibition of FSK/Src activity. Upregulation of ERK and mTOR signaling pathways.	
*Pkd1*	*Pkd1^F/RC^*	-	Increased methionine and S-adenosylmethionine (SAM).	[81]
	*Pkd1^RC/RC^*	Dietary restriction of methionine	Dietary restriction of methionine.	
	*Ksp-Cre; Pkd1^F/RC^; Mettl3^F/F^*	Deletion of Mettl3 gene, a key component of SAM (double mutant)	Delayed expansion of cysts.	
*Pkd1*	*KspCreERT2; Pkd1^lox/lox^; Tmem16a^lox/lox^*	TMEM16A gene (double mutant)	Inhibition of Ca^2+^ signaling pathway and cell proliferation. Increased CFTR expression.	[82]
	*Pkd1^fl/fl^; Cre/Esr1*,	Quantitative proteomics	Promotion of the Nuclear Factor Erythroblast 2-Related Factor 2 (NRF2) degradation.	
	*Pkd1^fl/fl^; Cre/Esr1; Nrf2* *^−^* *^/^* *^−^*	Deletion of NRF2 gene (double mutant)	Increased ROS generation. Inhibition of the cystic area expansion.	
**Gene**	**Strain/Gene Name**	**Treatment/Analysis/** **Administration**	**Results/Effects**	**References**
**Knock-in Mouse**				
*Pkd1*	*Pkd1^RC/null^*, *Pkd1^RC/RC^*	PKD1 targeted proteomic analysis	Reduction of TCA cycle, fatty acid oxidation, respiratory complexes, and endogenous antioxidants.	[62]
		Overexpression of mitochondria-targeted catalase (mCAT) using an adeno-associated virus vector	Reduction of mitochondrial Reactive Oxygen Species (ROS) and oxidative damage. Improvement of disease progression in PKD. Partial improvement in the TCA cycle and fatty acid oxidation.	
*Pkd1*	*Pkd1^RC/RC^*	Eramipretide, a mit ochondrial protective tetrapeptide	Attenuated ERK1/2 phosphorylation. Improved mitochondrial supercomplex formation. Improvement of PKD progression.	[63]
*Pkd1*	*Pkd1^RC/RC^*	Metformin	Suppression of PKD progression. Reduction of cell proliferation markers. Reduction of inflammation and injury markers.	[64]
*Pkd1*	*Pkd1^RC/RC^*	Lixivaptan, a novel Vasopressin Receptor 2 (V2R) antagonist and R-568, a calcium receptor agonist	Reduction of the cAMP levels. Suppression of the PKA activity. Reduction of phosphorylated AMPK and ERK. Suppression of PKD progression. Suppression of fibrosis.	[65]
*Pkd1*	*Pkd1^RC/RC^*	BLU2864, a selective PRKACA (AMP-dependent protein kinase) inhibitor	Inhibition of PKA activity. Inhibition of cyst formation, growth-promoting pathways, and cyst formation.	[66]
*Pkd1*	*Pkd1^RC/RC^*	VX-809, a modulator of CFTR trafficking and processing	Increased basolateral membrane co-localization of CFTR. Decreased HSP27. Inhibition of PKD progression.	[67]
*Pkd1*	*Pkd1^RC/RC^*	Nintedanib, a receptor tyrosine kinase (RTK) inhibitor	Suppression of PKD progression. Suppression of the cell proliferation. Reduction of the growth factor and fibrosis expressions.	[68]
*Pkd1*	*Pkd1^RC/RC^*	Administration of Extracellular Vesicle (EV)/exosomes, Increased expression of EV/exosomes	Promotion of cyst formation and fibrosis. Increased phosphorylation of AKT, S6, Rb, STAT3, ERK.	[83]
		GW4869 to inhibit exosome biogenesis/release	Suppression of cyst formation.	
*Pkd1*	*Pkd1^RC/RC^*	Targeted metabolomics approach	Alteration of the biosynthesis and metabolism of tryptophan and arginine. Increase of indoles, kynurenine, and polyamines.	[84]
**Genome Editing**				
*Pkhd1*	Pkhd1^C642*^	Genome editing	Heterozygous Pkhd1^C642*^ developed hepatic cysts. Homozygous Pkhd1^C642*^ developed congenital hepatic fibrosis, inflammation of the portal field, fibrosis manifestations.	[85]
*Nphp1*	*Nphp1^del2−20/del2−20^*	Genome editing	Renal cysts. Thickening of the tubular basement membrane. Retinal degeneration. Abnormal spermatogenesis.	[86]
		Using of AAV9 vectors	Partial rescue of both renal and retinal phenotypes.	

Reference numbers also serve as reference numbers in the text.

## Data Availability

Not applicable.
